# Supplementary material for: TiO2 nanoparticles affect the bacterial community structure and Eisenia fetida (Savigny, 1826) in an arable soil
Source: PeerJ. 2019 Jul 25;7:e6939. doi: 10.7717/peerj.6939 (PMC6661143; doi:10.7717/peerj.6939)
Supplement: Supplemental Information 1 [file peerj-07-6939-s001.pdf]

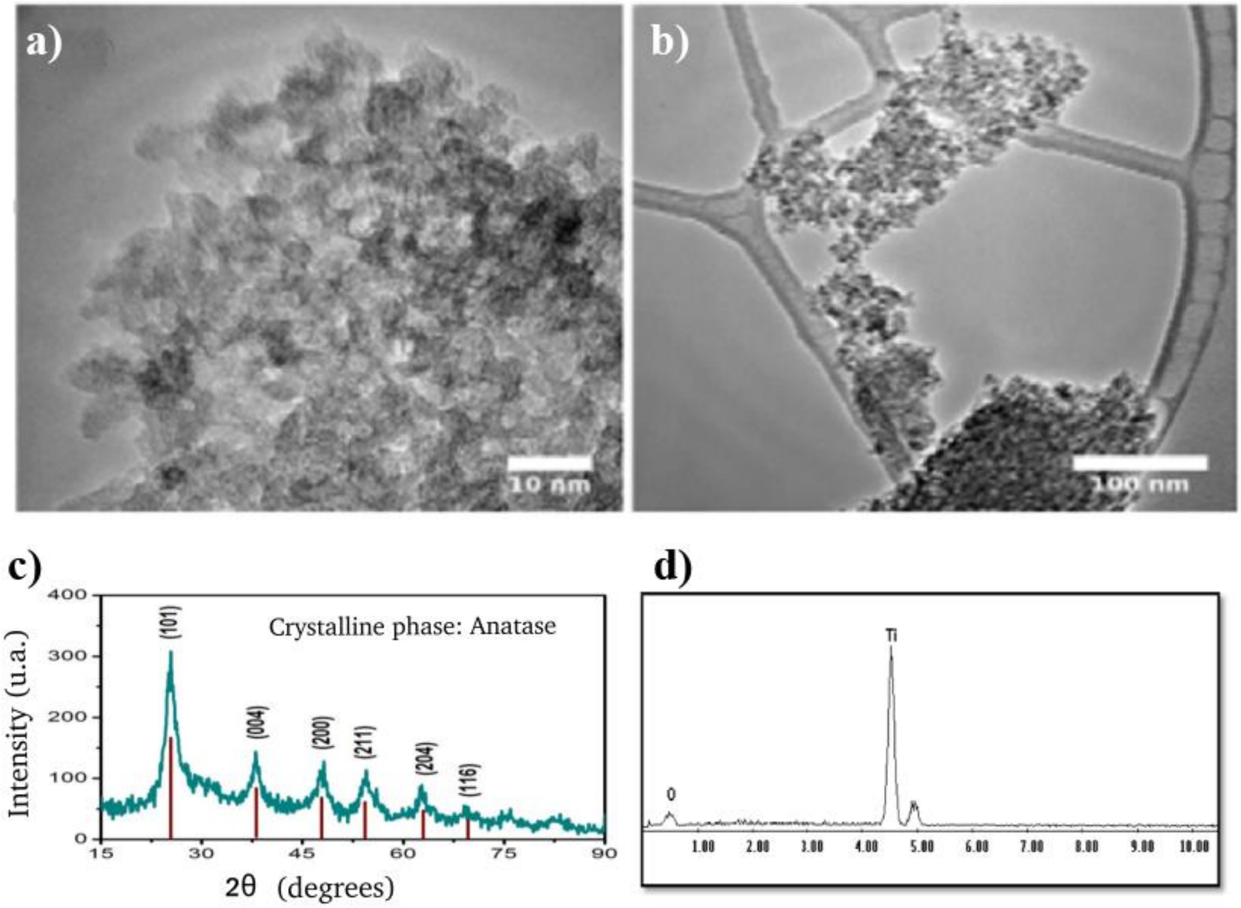


**Figure S1.** Pictures of the $TiO_2$ nanoparticles by a) and b) Transmission electron microscope (TEM), c) X-ray difraction (XRD) and d) Energy-dispersive X-ray spectroscopy (EDS).
