# Supplementary material for: TiO2 nanoparticles affect the bacterial community structure and Eisenia fetida (Savigny, 1826) in an arable soil
Source: PeerJ. 2019 Jul 25;7:e6939. doi: 10.7717/peerj.6939 (PMC6661143; doi:10.7717/peerj.6939)
Supplement: Supplemental Information 2 [file peerj-07-6939-s002.pdf]

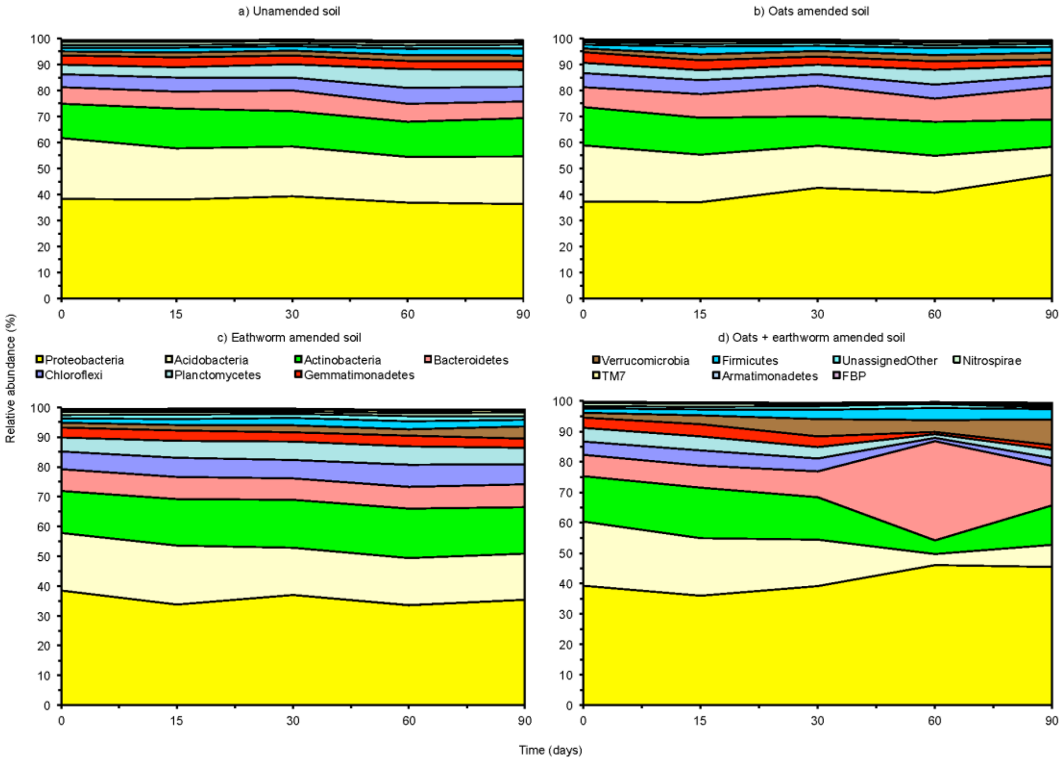

Figure S2. The relative abundance of the most abundant bacterial phyla in a) the unamended soil, b) soil amended with oats, c) soil amended with earthworms and d) soil amended with earthworms plus oats at the onset of the experiment and after 15, 30, 60 and 90 days.
