# Supplementary material for: TiO2 nanoparticles affect the bacterial community structure and Eisenia fetida (Savigny, 1826) in an arable soil
Source: PeerJ. 2019 Jul 25;7:e6939. doi: 10.7717/peerj.6939 (PMC6661143; doi:10.7717/peerj.6939)
Supplement: Supplemental Information 4 — a) Phyla and b) Genera. Non parametric analysis with * p < 0.05 and > 0.01, ** p ≤ 0.01 and > 0.001, and *** p ≤ 0.001. The bacterial genera were grouped according to the bacterial phyla, class or order they belonged to. The relative abundance of the bacterial groups (%) is given in the unamended soil (Unamended), while the changes in the relative abundance in soil amended with oats, earthworms or oats + earthworms is given as the ratio of (the relative abundance in the oats, earthworms or oats + earthworms amended soil)–(the relative abundance in unamended soil)/ (the relative abundance in the oats, earthworms or oats + earthworms amended soil). A red square means that the relative abundance of the bacterial group decreased and a blue square that it increased compared to the unamended soil. [file peerj-07-6939-s004.pdf]

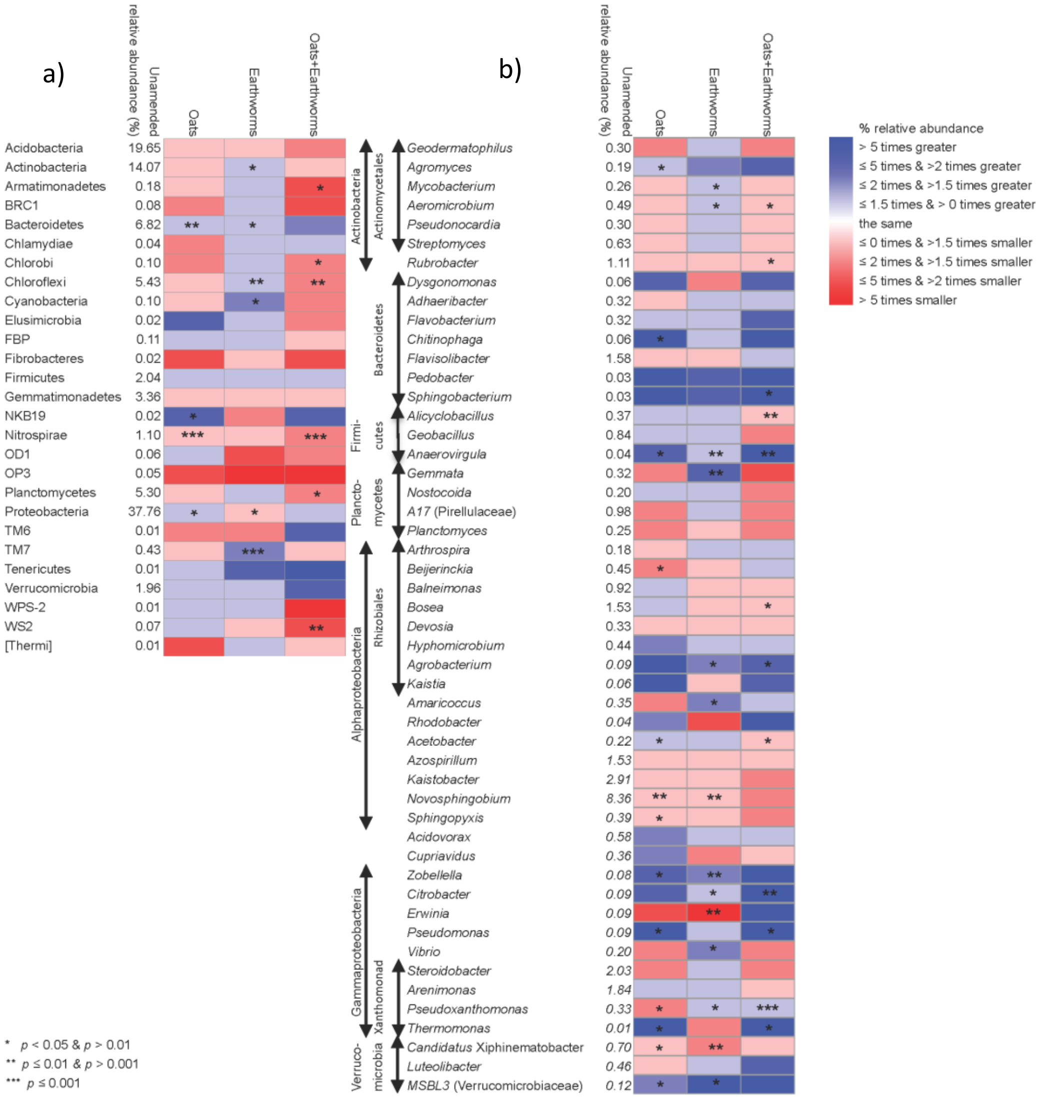

Figure S4. Heatmap with the effect of the application of oats, earthworms (*Eisenia fetida* (Savigny, 1826)) or oats plus earthworms on the relative abundance of bacterial phyla and 50 most abundant genera compared to the unamended soil (% relative abundance of the different bacterial groups). a) Phyla and b) Genera. Non parametric analysis with * $p < 0.05$ and > 0.01, ** $p \leq 0.01$ and > 0.001, and *** $p \leq 0.001$. The bacterial genera were grouped according to the bacterial phyla, class or order they belonged to. The relative abundance of the bacterial groups (%) is given in the unamended soil (Unamended), while the changes in the relative abundance in soil amended with oats, earthworms or oats + earthworms is given as the ratio of (the relative abundance in the oats, earthworms or oats + earthworms amended soil) – (the relative abundance in unamended soil)/ (the relative abundance in the oats, earthworms or oats + earthworms amended soil). A red square means that the relative abundance of the bacterial group decreased and a blue square that it increased compared to the unamended soil.
