# Supplementary material for: TiO2 nanoparticles affect the bacterial community structure and Eisenia fetida (Savigny, 1826) in an arable soil
Source: PeerJ. 2019 Jul 25;7:e6939. doi: 10.7717/peerj.6939 (PMC6661143; doi:10.7717/peerj.6939)
Supplement: Supplemental Information 5 [file peerj-07-6939-s005.docx]

Table S1. Analysis of variance Type III F test to determine the effect of treatment, nanoparticles and their interaction on soil characteristics after 90 days (aov, R Core team, 2014).

| ⎯⎯⎯⎯⎯⎯⎯⎯⎯⎯⎯⎯⎯⎯⎯⎯⎯⎯⎯⎯⎯⎯⎯⎯⎯⎯⎯⎯⎯⎯⎯⎯⎯⎯⎯⎯⎯⎯⎯⎯⎯⎯⎯⎯⎯⎯⎯⎯⎯⎯⎯⎯⎯⎯⎯ | | | | | | | | | | | | |
| --- | --- | --- | --- | --- | --- | --- | --- | --- | --- | --- | --- | --- |
|  | Organic carbon | | pH | | EC ^a^ | | WHC ^b^ | | NO_2_^-^ concentration | | NO_3_^-^ concentration | |
|  | ⎯⎯⎯⎯⎯⎯⎯ | | ⎯⎯⎯⎯⎯⎯ | | ⎯⎯⎯⎯⎯⎯ | | ⎯⎯⎯⎯⎯⎯⎯ | | ⎯⎯⎯⎯⎯⎯⎯⎯⎯ | | ⎯⎯⎯⎯⎯⎯⎯⎯⎯ | |
| Factor | F value | P value | F value | P value | F value | P value | F value | P value | F value | P value | F value | P value |
| ⎯⎯⎯⎯⎯⎯⎯⎯⎯⎯⎯⎯⎯⎯⎯⎯⎯⎯⎯⎯⎯⎯⎯⎯⎯⎯⎯⎯⎯⎯⎯⎯⎯⎯⎯⎯⎯⎯⎯⎯⎯⎯⎯⎯⎯⎯⎯⎯⎯⎯⎯⎯⎯⎯⎯ | | | | | | | | | | | | |
| Nanoparticles | 0.12 | 0.730 | 0.10 | 0.753 | 1.54 | 0.225 | 0.92 | 0.347 | 5.45 | 0.026 | 2.34 | 0.137 |
| Treatment | 1.23 | 0.317 | 10.21 | < 0.001 | 21.35 | < 0.001 | 0.48 | 0.696 | 12.92 | < 0.001 | 19.94 | < 0.001 |
| Interaction | 0.92 | 0.446 | 0.05 | 0.987 | 2.19 | 0.111 | 0.37 | 0.775 | 1.54 | 0.225 | 1.93 | 0.148 |
| ⎯⎯⎯⎯⎯⎯⎯⎯⎯⎯⎯⎯⎯⎯⎯⎯⎯⎯⎯⎯⎯⎯⎯⎯⎯⎯⎯⎯⎯⎯⎯⎯⎯⎯⎯⎯⎯⎯⎯⎯⎯⎯⎯⎯⎯⎯⎯⎯⎯⎯⎯⎯⎯⎯⎯ | | | | | | | | | | | | |

^a^ EC: Electrolytic conductivity, ^b^ WHC: Water holding capacity.

| ⎯⎯⎯⎯⎯⎯⎯⎯⎯⎯⎯⎯⎯⎯⎯⎯⎯⎯⎯⎯⎯⎯⎯⎯⎯⎯⎯⎯⎯⎯⎯⎯⎯⎯⎯⎯⎯⎯⎯⎯⎯⎯⎯⎯⎯⎯⎯⎯⎯⎯⎯⎯⎯⎯⎯ |
| --- |
