# Supplementary material for: TiO2 nanoparticles affect the bacterial community structure and Eisenia fetida (Savigny, 1826) in an arable soil
Source: PeerJ. 2019 Jul 25;7:e6939. doi: 10.7717/peerj.6939 (PMC6661143; doi:10.7717/peerj.6939)
Supplement: Supplemental Information 6 — a Mean from three substrate samples (n = 3), b value between brackets is standard deviation of the mean (n = 3), c Non parametric analysis (t1way analysis, WRS2 Package in R, Mair & Wilcox, 2017). [file peerj-07-6939-s006.docx]

Table S2. Effect of TiO_2_ nanoparticles on earthworm characteristics (*Eisenia fetida* (Savigny[, 1](https://en.wikipedia.org/wiki/Marie_Jules_César_Savigny)826)).

| ⎯⎯⎯⎯⎯⎯⎯⎯⎯⎯⎯⎯⎯⎯⎯⎯⎯⎯⎯⎯⎯⎯⎯⎯⎯⎯⎯⎯⎯⎯⎯⎯⎯⎯ | | | | | |
| --- | --- | --- | --- | --- | --- |
|  | | Earthworms amended soil | | | |
| Nanoparticles | ⎯⎯⎯⎯⎯⎯⎯⎯⎯⎯⎯⎯⎯⎯⎯⎯⎯⎯⎯⎯⎯⎯⎯⎯ | | | | |
| (mg kg^-1^ dry soil) | | Adult | Juvenile | Cocoons | Shells |
| ⎯⎯⎯⎯⎯⎯⎯⎯⎯⎯⎯⎯⎯⎯⎯⎯⎯⎯⎯⎯⎯⎯⎯⎯⎯⎯⎯⎯⎯⎯⎯⎯⎯ | | | | | |
| 0 | | 8.0 ^a^ (1.0) ^b^ | 1.3 (1.5) | 0.3 (0.6) | 1.3 (0.6) |
| 150 | | 5.3 (4.7) | 0.3 (0.6) | 0.0 (0.0) | 1.0 (1.0) |
| 300 | | 5.7 (2.1) | 0.7 (0.6) | 0.7 (0.6) | 0.3 (0.6) |
| *p* value | | 0.340 ^c^ | 0.611 | ND | 0.261 |
| ⎯⎯⎯⎯⎯⎯⎯⎯⎯⎯⎯⎯⎯⎯⎯⎯⎯⎯⎯⎯⎯⎯⎯⎯⎯⎯⎯⎯⎯⎯⎯⎯⎯ | | | | | |
|  | | Earthworms plus oats amended soil | | | |
| ⎯⎯⎯⎯⎯⎯⎯⎯⎯⎯⎯⎯⎯⎯⎯⎯⎯⎯⎯⎯⎯⎯⎯⎯⎯⎯⎯⎯⎯⎯⎯⎯⎯ | | | | | |
| 0 | | 9.7 (0.6) | 35.7 (18.5) | 8.0 (7.0) | 7.0 (5.3) |
| 150 | | 9.7 (0.6) | 62.0 (16.7) | 13.0 (1.0) | 7.3 (1.5) |
| 300 | | 10.3 (0.6) | 54.0 (17.5) | 12.3 (5.1) | 4.0 (2.6) |
| *p* value | | 0.405 | 0.331 | 0.598 | 0.340 |
| ⎯⎯⎯⎯⎯⎯⎯⎯⎯⎯⎯⎯⎯⎯⎯⎯⎯⎯⎯⎯⎯⎯⎯⎯⎯⎯⎯⎯⎯⎯⎯⎯⎯⎯ | | | | | |
| ^a^ Mean from three substrate samples (*n* = 3), ^b^ value between brackets is standard deviation of the mean (*n* = 3), ^c^ Non parametric analysis (t1way analysis, WRS2 Package in R, Mair and Wilcox, 2017). | | | | | |
| ⎯⎯⎯⎯⎯⎯⎯⎯⎯⎯⎯⎯⎯⎯⎯⎯⎯⎯⎯⎯⎯⎯⎯⎯⎯⎯⎯⎯⎯⎯⎯⎯⎯⎯ | | | | | |
